# Factors associated with disordered feeding among high school students in Kerman City, Iran

**DOI:** 10.1186/s40337-022-00559-0

**Published:** 2022-03-09

**Authors:** Abolfazl Dokhani, Mahlagha Dehghan, Masoud Rayani, Mahboobeh Maazallahi, Mansooreh Azzizadeh Forouzi

**Affiliations:** Nursing Research Center, Kerman University of Medical Sciences, Kerman, Iran

**Keywords:** Mental health, Disordered eating, Female student, Kerman

## Abstract

**Background:**

Mental health problems and disordered eating, are more common in adolescents. This study investigated relationship between mental health and disordered eating in high school girls in southeast Iran.

**Methods:**

This cross-sectional descriptive correlational study accomplished in high school girls of Kerman at the southeast of Iran in 2019. Using three parts demographic, Standard General Health Questionnaire (GHQ-28) and Eating Attitudes questionnaire, (Eat-26) with three subscales: eating habits, desire to eat and oral control. We investigated high school girl’s mental health and relationship with disordered eating with cluster sampling method (600 high school girl). Multivariate logistic regression was used to determine the association between significant variables and the risk of General Health (Yes/No) and Eating attitude (Yes/No). Spearman correlation test, Mann–Witheny U test and Kruskal–Wallis test were used, and Significant level was considered at *P* < 0.05.

**Results:**

A direct and significant relationship between mental health and disordered eating (r = 0/19, *P* < 0.001). In other words, the higher the mental health disorder score, the higher the disordered eating score, and the higher disordered eating score, the higher mental problems. There was a direct and significant relationship between mental health and all aspects of disordered eating including eating habits (r = 0/12, *P* < 0.05), desire to eat (r = 0/1, *P* < 0.05) and oral control (r = 0/14, *P* < 0.001).

**Conclusions:**

It seems that the disordered eating and mental health have a determinant role in relationship with each other. Therefore, prevention and health promotion programs should be implemented to improve female adolescent mental health and reduce disordered eating.

## Plain English summary

Mental health is considered one of the determinants of people's general health, and disordered eating is frequent in adolescents with the highest prevalence in adolescent girls. The present study describes the correlation between mental health and disordered eating in Iranian High School Girls. 600 participants in this study. There was a strong relationship between mental health and disordered eating, which means the higher the mental health disorder score, the higher the disordered eating score, and the higher disordered eating score, the higher mental problems. High school eating disorders and mental health should be monitored by public health and School health services (SHS) to provide interventions which focus on preventing this kind of problems.

## Background

Health is a multidimensional concept that includes happiness and well-being in addition to disease and disability [1]. Mental health is defined by World Health Organization as a state of well-being in which an individual recognizes his or her own potential, and uses it effectively and productively to cope with the normal stresses of life, can work productively and fruitfully, and is able to make a contribution to his or her community [2]. Mental health is important for maintaining good physical health and healthy lifestyle practices [3]. Mental health disorders in adolescence are a significant issue. Over the past two decades, there has been growing global concern over the prevalence of mental health problems amongst children and young people [4, 5]. Mohammadi et al. [6] reported psychiatric disorders such as conduct disorder (5.7%), attention deficit hyperactivity disorder (7.5%), generalized anxiety disorder (9.4%), obsessive–compulsive, social phobia, oppositional defiant disorder (10.1%), phobia (20.2%), and depression (16.4%) are the most common in children and adolescents with disorded eating.

Eating disorders are behavioral conditions characterized by severe and persistent disturbance in eating behaviors and associated distressing thoughts and emotions. They can be very serious conditions affecting physical, psychological and social function [7].

This disorder are characterized by serious disturbances in attitudes and behaviors around eating, including severe restriction of food intake and/or frequent binge eating and purging behaviors, such as self-induced vomiting and misuse of laxatives [8]. Eating disorders can affect both genders and people of all ages. However, according to the national institute of mental health, eating disorders primarily affect girls and women [9]. From a clinical point of view, EDs are an important cause of morbidity and mortality in adolescent girls and young adult women due to the severe changes in their eating behaviors [8, 9].

Eating disorders can cause individuals to experience a range of symptoms that can interfere with their ability to function there are many associated emotions that can lead to low self-esteem and difficulty participating socially [10].

Studies in Middle East such as Mollazadeh Esfanjaani et al. [11], reported based on the EAT-26 cut off score, 25.7% and 74.3% and of students were found with and without eating disorders, respectively. Anxiety was one of the factors associated with disordered eating in female students [12]. Also, in the study conducted in Iran by Mohammedi et al. [6], prevalence of feeding and disordered eating among children and adolescents was 0.89 (0.81–1.10) and psychiatric comorbidities were significantly more common compared to their peers without feeding and disordered eating [6]. Gargari et al. [13] reported 16.7% (C.I with 95%: 15.1–18.3%) of students had disordered eating attitudes [14]. Besides of Iran the prevalence of disordered eating attitudes among high-school girls of Al-Madinah in Saudi Arabia was 42.5% [15], in Kuwaiti adolescents was 47% [16], in Oman was 29.4% [17], Saudi Arabia 24.6% [18], Palestine 38.9% [19] and in the UAE 23.4% [17]. It has been estimated that up to 14% of all youth display disordered eating [20].

A strong positive relationship has been reported between risk of disordered eating and stressors such as academic overload, constant pressure to succeed, competition with peers and concerns about the future [21]. Adolescents who are susceptible to depression are found to be more vulnerable to developing disordered eating [22]. Weight and shape concerns, body dissatisfaction, perceived pressure to be thin and thin-ideal internalization are a precursor to disordered eating [23, 24].

Adolescence is a critical developmental period, and over-valuing beauty may be important for girls because of the role modeling by social media and cyberspace. Awareness of the psychology factors that may be associated with eating disorders or predispose students to these disorders seems necessary. According to the researcher’s information, no relational mental health and disordered eating studies have been carried out in this area in south-east of Iran Therefore, the present study hypothesis there is a relationship between mental health and disordered eating in high school girls in Kerman in south-eastern Iran.

## Methods

### Setting

This was a cross-sectional descriptive correlational study in which high school girls of Kerman City in southeast Iran participated in the study. High schools in Kerman are divided into two district. One public high school and one private high school were selected from each district (256 students), as well as from district two (344 students).

### Participants

Samples were selected using multistage cluster sampling. The sample size for this study was 300 through the following formula. Since it was a cluster sampling, the design effect was considered to be 2, and the number of samples increased to 600. In Iran, high school has three levels, where students between the ages of 16–18 are studying. This study was performed on female high school students in Kerman. Inclusion criteria for this study were living in Kerman, willingness to participate in research, participant have to be attending school. Persons who did not consent to participate, and incomplete questionnaires were excluded from the study. Participants who had inclusion criteria participated in the study.

$$n = \left[ {\frac{{z_{{1 - \frac{\alpha }{2}}} + z_{1 - \beta } }}{c}} \right]^{2} + 3$$

$$c = 0.5{\text{ln}}\left( {\frac{1 + r}{{1 - r}}} \right)$$, ($${\text{z}}_{{1 - \frac{\alpha }{2}}}$$  = 1.96, α = 0.05, $${\text{z}}_{{1 - {\upbeta }}}$$ = 0.84, r =  − 0.2).

The 95% confidence factor was calculated, so Z_1−α/2_ is 1.96.

The test power is 1 − β = 0.8 and Z_1−α/2_ = 0.84.

### Study procedure

After obtaining the ethics code, the first researcher visited the research setting. Permits were obtained from the Education and Security department. The Participants were selected based on the inclusion criteria. Informed consent was obtained before data collection. Students were ensured that their information would remain confidential and then the questionnaires were distributed. Data collection was lasted from May 2017 to June 2018.

## Measures

### Demographic characteristics form

Demographic characteristics form that included information such as weight, height, fathers and mother’s education level and occupation, family income, parental relationships, living with parents, number of sisters and brothers, birth rank, rate of exercise, disease history.

### GHQ-28

The General Health Questionnaire (GHQ-28) was designed and validated by Goldberg and Hillier [25]. This 28-item self-reporting questionnaire requests participants to indicate how their health in general has been over the past few weeks. The Likert Scale 0,1,2,3 scoring, used in the original and current studies, indicated the following frequencies of experience: not at all, no more than usual, rather more than usual and much more than usual. The GHQ-28 was divided into four subscales: physical symptoms (items1–7), anxiety symptoms and sleep disorder (items 8–14), social function (items 15–21) and depression symptoms (items 22–28). The minimum score for the 28-question version was 0, and the maximum was 84. Higher GHQ-28 scores indicate higher levels of distress. Scores from 0 to 22 indicate no, 23 to 40 mild, 41 to 60 moderate, and 61 to 84 severe on the whole scale indicate symptoms of morbidity. The Persian version of the questionnaire was validated by Nazifi et al. in Iran [26]. The questionnaire reliability was considered to be 0.92 by estimating Cronbach’s alpha Coefficients. The questionnaire also had suitable content validity [26].

### EAT-26

The Eating Attitude Questionnaire is widely used as a self-assessment tool for disordered eating attitudes and behaviors. This 26-item questionnaire consists of 3 subscales: eating habits, desire to eat and oral control. Eleven items of questionnaire measure the nutrition attitude, while 15 items are related to diet practice. The responses in EAT-26 are scored on a Likert scale: Never = 0, Rarely = 0, Sometimes = 0, Often = 1, Very Often = 2, and Always = 3. A score equal to 20 or greater is defined as a disordered eating attitudes. In Iran, reliability and validity of the translated EAT-26 were 0.80, 0.76, respectively in the study conducted by Gargari et al. [13].

### BMI

Body mass index (BMI) was calculated from self-reported Height and weight., BMI = (kg/m^2^) [27].

### Statistical analysis

Data were analyzed by SPSS 20 version. Frequency, percent, mean, and standard deviation were used to describe demographic characteristics. Kolmogorov–Smirnov test was used to study normalization of quantitative variables. Mental health and disordered eating scores did not have normal distributions. Therefore, Spearman correlation coefficient, Mann–Witheny U, and Kruskal–Wallis tests were used. Multivariate logistic regression was used to determine the association between significant variables and the risk of General Health (Yes/No) and Eating attitude (Yes/No). The significant level was considered at *P* < 0.05.

## Results

Findings showed a direct and significant relationship between mental health and disordered eating (r = 0.19, *P* < 0.001). There was a direct and significant relationship between mental health and all aspects of disordered eating including eating habits (r = 0/12, *P* < 0.05), desire to eat (r = 0.1, *P* < 0.05) and oral control (r = 0/14, *P* < 0.001). Dimensions of physical symptoms, anxiety and sleep disorder, and depression were directly and significantly related to disordered eating and all its dimensions. Depression had a direct and significant relationship to disordered eating, eating habits and oral control. There was no significant relationship between social functioning, disordered eating and the dimensions (*P* > 0.05) (Table 1).

**Table 1:** The Relationship between Mental health, eating disorder and demographic characteristics (qualitative variables) of subjects

Variable		Frequency (%)	Mental health		Eating disorders	
			Mean/SD	Statistic test/*P* value	Mean/SD	Statistic test/*P* value
Maternal education	Uneducated	11(2)	39(7.91)	λ^2^ = 3.44(0.49)	16.27 (14.06)	λ^2^ = 1.83(0.77)
	Middle school	73(13.5)	34.41(14.62)		13.68(8.65)	
	Diploma	294(54.3)	35.14(14.5)		13.14(8)	
	Bachelor of art	123(22.7)	33.55(14.12)		14.44(8.62)	
	Master and higher	40(7.4)	37.4(16.4)		14.8(9.27)	
Paternal education	Uneducated	9(1.7)	39.11(11.99)	λ^2^ = 6.01(0.2)	17.88(11.46)	λ^2^ = 1.98(0.74)
	Middle school	80(15)	35.88(14.59)		13.65(8.4)	
	Diploma	276(51.7)	34.68(14.06)		13.58(8.39)	
	Bachelor of art	119(22.3)	33.27(15.27)		13.45(8.3)	
	Master and higher	50(9.4)	37.96(14.87)		15.06(9.52)	
Mother’s job	Housewife	376(70)	34.17(14.07)	Z = − 1.6(0.11)	13.74(8.54)	Z = − 0.08(0.94)
	Employed	161(30)	36.55(15.25)		13.77(8.22)	
Father’s job	Unemployed	27(5.2)	34.51(14.92)	Z = − 0.001(0.99)	13.96(9.32)	Z = − 0.14(0.89)
	Employed	494(94.8)	34.61(14.42)		13.65(8.46)	
Income	< 500,000 tomans	31(6)	35.12(12.35)	λ^2^ = 0.82(0.84)	13.61(9.34)	λ^2^ = 0.64(0.89)
	500,000–1,000,000 tomans	82(15.9)	34.14(16.25)		14.2(8.38)	
	1,000,000–1,500,000	107(20.7)	34.56(13.91)		14.51(9.77)	
	> 1,500,000	296(57.4)	35.24(14.48)		13.44(8.22)	
Parental relationship	Living with each other	505(96)	3. 21(14.24)	Z = − 3.41(0.001)	13.7(8.56)	Z = − 0.1(0.32)
	Got divorced	21(4)	46.57(16.52)		15.61(8.89)	
Living with parents	With father or mother	4(0.8)	41.06(16.22)	Z = − 2.43(0.02)	15.97(10.67)	Z = − 0.86(0.39)
	Both	478(93)	34.46(14.31)		13.64(8.28)	
Number of sisters	None	208(38)	34.39(14.24)	λ^2^ = 0.84(0.84)	13.36(7.94)	λ^2^ = 0.24(0.97)
	One	225(41.1)	35.63(14.88)		13.95(8.82)	
	Two	86(15.7)	34.45(14.38)		13.16(8.48)	
	Three and more	28(5.2)	34.32(13.76)		14.32(10.59)	
Number of brothers	None	212(38.9)	34.68(15.02)	λ^2^ = 2.4(0.49)	13.87(9.02)	λ^2^ = 1.04(0.97)
	One	229(42)	34.76(14.57)		13.32(7.89)	
	Two	81(14.9)	36.76(14.07)		14.24(8.61)	
	Three or more	23(4.2)	32.26(9.47)		14.95(9.86)	
Birth rank	First	247(47.8)	34.31(14.26)	λ^2^ = 1.76(0.62)	14.18(8.42)	λ^2^ = 3.28(0.35)
	Second	146(28.2)	36.43(14.64)		13.15(9.3)	
	Third	79(15.3)	34.16(14.94)		12.45(6.95)	
	Fourth and higher	45(8.7)	34.75(13.64)		13.75(7.55)	
Rate of exercise	Not at all	101(18.7)	40.64(14.37)	λ^2^ = 25.37(< 0.001)	14.53(8.18)	λ^2^ = 6.14(0.19)
	Very low	23(3.4)	35.82(13.73)		13.43(6.74)	
	Low	198(36.7)	34.88(14.2)		12.7(7.61)	
	Moderate	170(31.5)	32.6(14.34)		13.56(9.07)	
	Intense	47(8.7)	30.53(13.4)		15.57(8.72)	
Disease history	No	484(88.5)	33.79(14.22)	Z = − 4.88(< 0.001)	13.53(8.49)	Z = − 1.43(0.15)
	Yes	63(11.5)	43.44(13.69)		15.17(8.67)	
Variable		Mean/SD				
BMI		17.02(3.2)		R = − 0.05(0.27)		r = 0.09(0.03)

r = Spearman correlation coefficient, Z = Mann–Whitney U test, λ^2^ = Kruskal Wallis test

According to the GHQ cut point, 23.9% (n = 131) participants had no symptoms of psychological disorder, while 76.1% (n = 416) had mild to severe symptoms of psychological disorder. The bivariate analysis showed GHQ score had a significant relationship with parental relationship, living with parents, rate of exercise, and disease history (*P* < 0.05) (Table 2). Multivariate logistic regression with backward method was conducted for further analysis. All variables with *P*-value < 0.2 in bivariate analysis included in the multivariate logistic regression model (i.e., Eating Attitude Questionnaire score, mother’s job, parental relationship, living with parents, rate of exercise, and disease history). The results showed that rate of exercise, and disease history were significantly associated with psychological disorder, the risk of psychological disorders was 0.49 (95% CI for odds ratio: 0.25–0.96, *P* = 0.04), 0.42 (95% CI for odds ratio: 0.21–0.83, *P* = 0.01), 0.38 (95% CI for odds ratio: 0.16–0.93, *P* = 0.04) less in participants who exercised low, moderate, and intense respectively than those who did not exercised at all. In addition, the risk of psychological disorders was 2.55 (95% CI for odds ratio: 1.05–6.19, *P* = 0.04) times higher in participants who had disease history, than those who did not have.

**Table 2:** The relationship between mental health, its dimensions, eating disorder and its dimension in high-school girls

Variable	Eating disorder		Eating habits		Desire to eat		Oral control	
	ρ	*P*-value	ρ	*P*-value	ρ	*P*-value	ρ	*P*-value
Mental health	0.19	< 0.001	0.12	0.004	0.1	0.01	0.14	< 0.001
Physical symptoms	0.22	< 0.001	0.15	< 0.001	0.14	< 0.001	0.15	< 0.001
Anxiety and sleep disorder	0.23	< 0.001	0.16	< 0.001	0.12	0.005	0.17	< 0.001
Social function	− 0.06	0.13	− 0.06	0.16	− 0.37	0.39	− 0.06	0.17
Depression	0.13	0.002	0.09	0.03	0.05	0.2	0.09	0.03

ρ = Spearman correlation coefficient

According to the Eating Attitude Questionnaire cut point, 18.1% (n = 99) participants had disordered eating. The bivariate analysis showed Eating Attitude Questionnaire score had a direct and significant relationship with weight and body mass index (*P* < 0.05) (Table 2).

Multivariate logistic regression with backward method was conducted for further analysis. The results showed that GHQ score and BMI were significantly associated with disordered eating. Participants with disordered eating has lower general health and higher BMI than those without disordered eating (Table 3).

**Table 3:** The logistic model of the associations between important variables and disordered eating

Variable*	Multivariate logistic regression		
	Odds ratio	95% Confidence interval for odds ratio	*P* value
General health questionnaire score	1.02	1.002–1.03	0.02
Body Mass Index	1.10	1.02–1.17	0.008

*Rate of exercise and disease history also were included in the logistic model with backward method, however none of them remained in the final model

## Discussion

The results showed a direct and significant relationship between the mental health and disordered eating (*P* < 0.001), which support our hypothesis. This result is in consistence with the study results of Manaf et al. [28], Soleymani et al. [29], Mollazadeh Esfanjaani et al. [11], Maguen et al. [30], Micali et al. [31] and Field et al. [14], Touchette et al. [30], Mohammadi et al. [6]. The results of this study and literature reviews show that there is a relationship between general health and eating disorder attitudes in high school girls in Western and Eastern countries. Despite the use of different and identical questionnaires, this difference was significant. During adolescents, many changes take place physically and mentally. Co-occurring mental health diagnosis can begin around the same time as an eating disorder, can precede it, or can emerge after the eating disorder has already begun. Mood and anxiety disorders most commonly occur alongside eating disorders [32].

The findings demonstrated there was a direct and significant relationship between mental health dimensions (depression, physical symptoms, anxiety and sleep disorder), disordered eating, and disordered eating and all its dimensions (eating habits, oral control and desire to eat). There was no significant relationship between social functioning, disordered eating attitudes, and all its dimensions (*P* > 0.05). The association between most aspects of mental health and disordered eating indicates that the social function dimension has been unaffected. Consistence with this finding, study result of Mollazadeh Esfanjaani et al. [11] also reported no significant difference with disordered eating and social functioning domain of mental health [11]. In inconsistency with this result, Patel et al. [33] believed adolescents with disordered eating reported social difficulties. Chavez and Insel [34] claimed disordered eating often co-occur with psychiatric disorders and disturbances, including depression, anxiety, obsessionally, substance abuse disorders, and marked impairments in social functioning. Differences in study results can be due to cultural context. Lansford et al. [35] clamied children and adolescents grow up in a culture, and their behavior, values, social relationships, ways of seeing the world, language, and thought processes cannot be understood separate from culture [35]. Patel et al. [33] believe as adolescence is a unique stage in human development and typical brain maturation at this point in the lifespan is proposed to result in a sensitive period in adapting to one’s social context, leading to difficulties with social cognition.

Result related to the relationship between mental health and demographic characteristics showed that students whose parents were not divorced, who were living with both parents, and had no history of disease, had better mental health. There were no similar or diverse studies in the literature review. It has to be mentioned families in Iran are nuclear families and children live with their families until old age, even during university and before marriage.

It can be said living with parents, having no illness and no experience of divorce, provides less problems for participants. On the other hand, further studies in this field need to carry out.

Finding of the study showed students who exercised at less, moderate, or high levels had better mental health. This is in agreement with Costigan et al. and Wegner et al. [36, 37] they conclude exercise improved cognitive and mental health outcomes in adolescent populations and enhances brain structure and performance via direct and indirect physiological, cognitive, emotional, and learning mechanisms. Soltanian et al. [38] reported sport programs could help adolescents mental health and improve their problem-solving skills and enhance their coping strategies with mental health problems, and even to prevent the onset of psychological symptoms.

The present study showed the Body Mass Index was higher in participants with disordered eating than those without disordered eating that was in agreement with other studies [39, 40]. Also in agreement with the present study Yilmaz et al. [39] reported observing children whose BMI trajectories persistently and significantly deviate from age norms for signs and symptoms of ED could assist the identification of high-risk individuals. This study has some limitations. One of the limitations of the present study is the cross-sectional nature of this study. Self-reporting nature of the questionnaire could be the other limitation. A causal relationship cannot be determined. Based on the results future interventional studies are recommended to promote or increase mental health and decrease disorder eating. Study of other predicators of mental health and disordered eating also could be helpful. Using effective interventions could be effect on both mental health and eating disorders attitudes.

## Conclusion

According to the results of the present study, there was a direct and significant relationship between mental health and disordered eating. A Multi-dimensional school program which target mental health and disorder eating would be helpful to improve mental health and mange or prevent disordered eating. Awareness of appropriate nutrition in relation to body weight is needed among high school students. Community health nurses who have sufficient knowledge of school health can be helpful in this regard.

## Data Availability

The data set is available from the corresponding author upon reasonable request.

## Abbreviations

GHQ: General Health Questionnaire
SHS: School health services
EAT: Eating attitude tool
BMI: Body Mass Index
CKD: Chronic kidney disease
ED: Eating disorders
